# Two Useful Things

**Published:** 1872-03

**Authors:** Alex Warner

**Affiliations:** St. Clair City, Mich.


### TWO USEFUL THINGS.

BY ALEX. WARNER, D. D. S., ST. CLAIR CITY, MICH.

No. 1. To take an impression of soft gums, take two bowls, one with plaster enough to fill the impression cup, and one with enough to cover the soft parts of the mouth. Lay a napkin on the tongue of the patient and hold back the lips and cheeks; wipe the gums dry, and with a spatula cover the soft parts of the mouth. When this is sufficiently set fill the impression cup and place in the mouth over that first introduced. When the latter is taken away a perfect impression will be the result.

No. 2. If you havn't a dollar to buy a punch for Barnum's Rubber Dam (Missouri Dental Journal, Vol. III Journalistic says Dr. Colburn does not use the rubber dam, because he gets along perfectly well without it. We can understand very well how this getting along perfectly well without it is accomplished. It generally indicates that the operator is accustomed to going around difficulties rather than surmounting them. We have in view such a getting along a clear appreciation of doctrines and practices that do not mark the skillful and honest practitioners of this day, and we imagine cases where amalgam is better than gold, and these cases are liable to be found wherever there is much difficulty in filling with gold. Such practitioners are sure to degenerate into first-class tooth carpenters, and the quicker they understand that this is the inevitable tendency, and reform their practice the better for themselves as well as for their patients). Just take a bur or the round end of any instrument and stretch the rubber over it until a round piece flies out, which it will surely do. By using different sized burs, holes of different sizes can be produced.

---

LAWRENCE, Jan. 6, 1872.

DR. TAFT, Editor of the DENTAL REGISTER—Dear Sir: In the December number of the REGISTER, which I received lately, is a brief paper by W. E. Driscoll, in which I think unkind and unnecessary judgment is given of the Kansas State Dental-Society.

The meeting of our Society in November was its first meeting after organization. At the meeting for organization in May, 1871, it was not possible to organize exercises for the November meeting, chiefly because so few were present, and the November meeting in reality was a meeting completing the organization, and the discussion held was intended merely to acquaint each other with the general practice which was observed by members. The discussion was entirely irregular and altogether practicable as was intended on account of no order being established for discussion or essays on subjects of interest to the profession. At our next the proceedings are expected to compare favorably even with the Lake Erie Association.

When Dr. Driscoll says that other societies are investigating questions which it seems "our Kansas brethren have not as yet heard of" he is certainly assuming and judging unnecessarily, if not unkindly.

When he speaks of our Kansas brethren not having "*our advantages*" it certainly seems as if he meant "OUR ABILITIES," for, if he is acquainted with the educational facilities that Kansas affords, and the means our country has for the diffusion of knowledge of all kinds, he will certainly see that he can not mean "advantages." Dr. D. further says: "We might compare numerous passages from each of these reports, but shall refrain so that every one may do so for himself," thus leading the reader to think that the report of the Kansas Society contained some assertion ignorant or ungrammatical. It certainly would have been better to give the extracts and comparisons in his paper. Evidently Dr. D. thinks Kansas is "outside" and so writes, but I hope he will make it convenient to attend our next meeting and we will take pleasure in welcoming him and dispelling his illusion. Hoping, Mr. Editor, you will give this space in the REGISTER,

I am very respectfully,

J. D. PATTERSON, Recording Secretary,
Kansas State Dental Society.

